# Continuous Synthesis of Double-Walled Carbon Nanotubes with Water-Assisted Floating Catalyst Chemical Vapor Deposition

**DOI:** 10.3390/nano10020365

**Published:** 2020-02-20

**Authors:** Liyu Dong, Jin Gyu Park, Branden E. Leonhardt, Songlin Zhang, Richard Liang

**Affiliations:** 1 High-Performance Materials Institute (HPMI), Florida State University, 2005 Levy Ave., Tallahassee, FL 32310, USA; ld15c@my.fsu.edu (L.D.); bel14c@my.fsu.edu (B.E.L.); sz15c@my.fsu.edu (S.Z.); zliang@fsu.edu (R.L.); 2 Materials Science and Engineering, 2005 Levy Ave., Tallahassee, FL 32310, USA; 3 Department of Industrial and Manufacturing Engineering, FAMU-FSU College of Engineering, 2525 Pottsdamer St., Tallahassee, FL 32310, USA; 4 Department of Chemical and Biomedical Engineering, FAMU-FSU College of Engineering, 2525 Pottsdamer St., Tallahassee, FL 32310, USA

**Keywords:** double-walled carbon nanotube (DWCNT) synthesis, water-assisted FCCVD, electrical conductivity, nano-indentation, anisotropic ratio

## Abstract

Double-walled carbon nanotubes (DWCNTs) were synthesized and continuously collected using a water-assisted floating catalyst chemical vapor deposition (FCCVD) method. Differing from the conventional water-assisted synthesis in which water vapor is one part of the carrier gas mixture, we included de-ionized water in the catalyst system, which achieved a more uniform and controlled distribution for efficient DWCNT production. Using a water-assisted FCCVD process with optimized conditions, a transition from multi- to double-walled CNTs was observed with a decrease in diameters from 19–23 nm to 10–15 nm in tandem with an elevated Raman I_G_/I_D_ ratio up to 10.23, and corroborated from the decomposition peak shifts in thermogravimetric data. To characterize the mechanical and electrical improvements, the FCCVD-CNT/bismaleimide (BMI) composites with different water concentrations were manufactured, revealing high electrical conductivity of 1720 S/cm along the bundle alignment (collection) direction, and the nano-indentation tests showed an axial reduced modulus at 65 GPa. A consistent value of the anisotropic ratio at ~3 was observed comparing the longitudinal and transverse properties. The continuous capability of the presented method while maintaining high quality is expected to result in an improved DWCNT mass production process and potentially enhance the structural and electrical applications of CNT nanocomposites.

## 1. Introduction

Since the discovery of carbon nanotubes (CNTs) in 1991 [1], CNTs have been studied intensively because of their exceptionally high mechanical [2,3], electrical [3], and thermal properties. However, transferring these properties into bulk products requires the development of efficient and reliable CNT synthesis methods. Particularly, the continuous synthesis and growth of long CNTs have become crucial challenges. Historically, arc-discharge [4] and laser ablation [5] have been used, but the carbon evaporation from solid targets requiring temperatures as high as 3000 °C has been proven impractical due to the high energy consumption and inefficiency. The conventional chemical vapor deposition (CVD) synthesis method [6] relies on the carbon decomposition on a pre-deposited substrate with transition metal catalyst (e.g., Fe, Co, and Ni). Requiring remarkably lower synthesis temperatures (700–1300 °C), the CVD method has been broadly used in laboratory and industrial environments. 

However, CVD-grown CNTs suffer from the carbonaceous and metallic impurities, possibly due to the relatively low catalytic efficiency. For the purpose of purification, complicated post-treatment techniques, including chemical oxidation [7,8,9] and physical separation [10,11], have been developed. Hata et al. reported a novel synthesis method in which they introduced a controlled amount of water [12,13]. In this process, the water vapor served as a weak oxidizer and selectively removed the amorphous carbon without damaging the CNT network, resulting in vertically aligned single-walled CNT forests with a purity above 99.98%. In a more recent work [14], the kinetics of super-growth CVD were probed through a quantitative time-evolution analysis, with the catalyst activity treated as radioactive decay. With a prolonged catalyst lifetime, the water-assisted CVD technique is anticipated to provide a simplified approach for achieving high-purity CNT synthesis more efficiently, potentially unlocking new CNT-related research developments and applications.

While the conventional substrate-based CVD can tune the nanotube diameters by precisely controlling the catalyst sizes, significant batch-to-batch variations remain a drawback. On the other hand, the floating catalyst chemical vapor deposition (FCCVD) [2,15,16,17,18] technique is attracting attention from both academia and industry. With the appropriate choice of reactants, precise condition control, and continuous winding collection of the CNT aerogel, the FCCVD process could achieve flexibility in product forms (e.g., yarns, sheets) providing mass production potential [19]. 

To further improve the quality and properties of the FCCVD-CNTs, de-ionized water was added as the cleaning agent. Water-containing catalyst was injected simultaneously into the hot zone at 1250 °C with carrier gas flow composed of H_2_ and Ar, and the effects of water on quality and properties of the resultant CNTs were analyzed and compared to the control group without water assistance.

## 2. Materials and Methods

**Figure 1:**
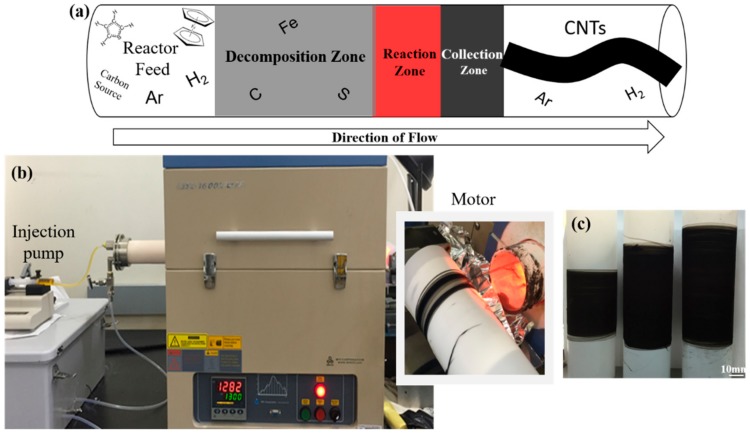
Floating catalyst chemical vapor deposition (FCCVD) synthesis setup: (**a**) schematic illustration of the High-Performance Materials Institute (HPMI) in-house reactor, (**b**) experimental set-up, and (**c**) the as-synthesized floating catalyst chemical vapor deposition carbon nanotubes (FCCVD-CNTs).

The catalyst solution was composed of ferrocene (98%, Sigma-Aldrich, St. Louis, MO, USA, used as received), thiophene (≥99%, Sigma-Aldrich, St. Louis, MO, USA, used as received), acetone as the carbon source, and de-ionized water as the oxidizer. The standard catalyst solution consisted of the Fe:S molar ratio set at 1:3, and 0, 1, and 2 mL de-ionized water was added in order to investigate the effects of water concentration, denoted as CNT-0, -1, and -2, respectively. The solution was injected into a close-ended alumina tube reactor (60 mm (O.D.) × 56 mm (I.D.) × 1000 mm (L)) set at 1250 °C and carried through by a flowing gas mixture of ultra-high purity (UHP) argon and hydrogen at the flow rates of 700–1200 sccm and 50–400 sccm, respectively. The liquid injection rate was closely monitored and adjusted within the range of 5–25 mL/h to maintain a continuous CNT aerogel collection. At the other end of the reactor, the aerogel-like CNT socks formed and aggregated on the inside wall before being collected continuously onto a rotating mandrel covered with a Teflon sheet, which served as the sample holder, as shown in Figure 1. For safety reasons, the exhaust gas, which is a mixture of high-concentration argon and hydrogen with some oxygen, had to pass through a one-way gas shield before venting into the atmosphere [20]. To keep consistency among the CNT-0, -1, and -2 groups, one plastic syringe (10 mL capacity) of catalyst solution was consumed for sample collection. A diluted bismaleimide (BMI)/acetone solution (0.1 wt.%–10 wt.%) was sprayed to fabricate the CNT/BMI composites, which were then cured at 191 °C for 4 h under vacuum, followed by a post-cure process of 227 °C for another 6 h. 

Scanning electron microscopy (SEM, JSM-7401F, JEOL, Tokyo, Japan) was used for morphological analysis, and EDAX Genesis XM4 spectroscopy (AMETEK Inc., Mahwah, NJ, USA) was used in conjunction to perform the energy-dispersive X-ray spectroscopy (EDS). The as-produced CNT quality was examined using a high-resolution transmission electron microscope (HRTEM, JEM-ARM200cF, JEOL, Tokyo, Japan), and the catalyst residue was measured by the thermogravimetric analysis (TGA, Q50, TA instruments, New Castle, DE, USA). The temperature ramping rate of TGA tests was 10 °C/min to 900 °C in air, and the specimens were taken and characterized at 600 °C, 700 °C, and 800 °C to study the oxidation resistance of CNTs and clarify the effects of catalyst cleaning. Raman spectra were collected using a Renishaw inVia confocal micro-Raman system with 0.5 mW laser power set at 785 nm excitation wavelength. A dynamic mechanical analysis (DMA, Q800, TA Instruments, New Castle, DE, USA) was used to characterize the tensile properties at room temperature, with the force ramping at 1 N/min. Tensile specimens were cut into 30 mm × 2 mm pieces with 15 mm gauge length, and five or more specimens were tested in each group in both directions—parallel and transverse to the CNT collection direction. Electrical conductivities were measured using a current source (Keithley 6221, Solon, OH, USA) and nanovoltmeter (Keithley 2182A, Solon, OH, USA) in a four-probe configuration. 

To study the effects of CNT quality on nanocomposite performance, CNT/carbon fiber (CF)/resin hybrid composites were fabricated, and nano-indentation tests were performed to measure the mechanical properties. The as-collected CNT sheets were condensed with acetone spray, saturated in a diluted BMI/acetone solution, and dried to produce the FCCVD-CNT/BMI prepregs. Unidirectional CF/BMI prepregs were cut into appropriately the same sizes, and the FCCVD-CNT/BMI prepregs were sandwiched in between, with the alignment direction parallel to each other. This assembly was then bagged under a full vacuum to expel air and cured using a similar process as previously reported [21]. Nano-indentation tests were performed on the mechanically polished samples along the longitudinal and transverse directions using an iNano® Nanoindenter (KLA-Tencor, Nanomechanics Inc., Oak Ridge, TN, USA) at the maximum load (50 mN). Ten or more indentations were performed with a Berkovich probe for each sample, and the load–displacement curves were analyzed using the Oliver–Pharr method [22,23].

## 3. Results and Discussion

**Figure 2:**
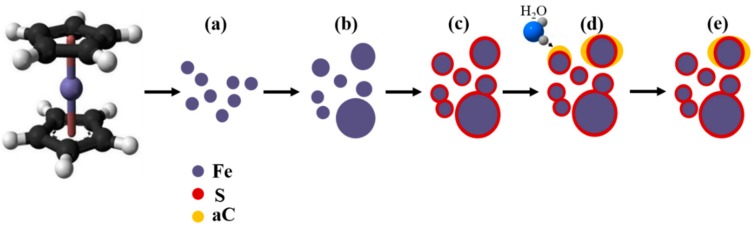
Iron catalyst formation and evolution mechanism: (**a**) ferrocene decomposition; (**b**) Fe nanoparticle agglomeration; (**c**) S coating formation; (**d**) amorphous carbon (aC) formation; (**e**) the aC removal induced by the water cleaning, which reactivates the catalyst nanoparticles.

One of the most widely postulated FCCVD synthesis mechanisms begins with the iron-containing organometallic precursor (i.e., ferrocene) decomposing at high temperatures to release iron atoms, which undergo agglomerations and form nanoparticles (Figure 2a) [24,25]. Inter-particle collisions and agglomerations result in various sizes of Fe nanoparticles (Figure 2b). The sulfur atoms, released from the thermal decomposition of thiophene, bond and coat the Fe nanoparticles on the surface (Figure 2c), which inhibit further catalyst particle growth. The sulfur-coated iron nanoparticles actively promote the continuous decomposition and pyrolysis of hydrocarbon species and initiate the growth of carbon nanotubes [15]. Amorphous carbon (aC) has been deemed a major byproduct of hydrocarbon pyrolysis [26], and its deposition on the surface of the catalyst particles renders them inactive. As experimentally verified by Yamada et al. using ‘Ball-CVD’ [27], water selectively reacted with the amorphous carbon, and the coating removal revived the catalyst activity, which considerably elevated the synthesis efficiency. Moreover, smaller-sized CNTs could be achieved as a result of the catalyst size shrinkage [28].

### 3.1. Oxidation Resistance

**Figure 3:**
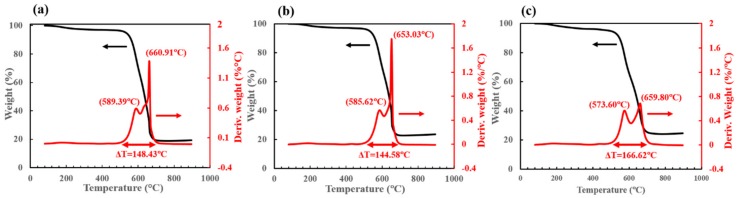
Thermogravimetric analysis results of the as-produced FCCVD-CNTs (tested in air): (**a**) CNT-0, (**b**) CNT-1, and (**c**) CNT-2.

To study the role of water in the FCCVD synthesis process, the quality of the as-produced CNTs from both the control (no water) and water-containing catalyst systems were compared. The as-produced CNTs were composed of various carbonaceous components, and their oxidation reactivities follow the order of amorphous carbon (aC) > single-walled nanotubes (SWCNTs) > double-walled nanotubes (DWCNTs) > multi-walled nanotubes (MWCNTs) [29,30]. Figure 3 shows the TGA results of the all three groups, with the temperatures of respective peaks labelled. With the increasing water concentration, the first derivative weight peak (shown in red) was observed to shift to a lower temperature—from 589.39 °C to 585.62 °C, and 573.60 °C, indicating the increasing amount of higher-curvature (possibly smaller-diameter) CNTs because of their higher reactivity with oxygen [31]. With the addition of de-ionized water, the second derivative weight peak at approximately 650–660 °C, which referred to the oxidation of the MWCNTs as another component, shifted left and shortened the temperature difference in between. A possible explanation is that the wall counts of MWCNTs were significantly reduced because of the catalyst cleaning effect, increasing the oxidation reactivity, thus yielding a higher weight derivative peak. With even higher water concentration (CNT-2), two derivative weight peaks of similarly lower intensities occurred, which may suggest the transition to more homogeneous CNT sizes and wall counts.

**Figure 4:**
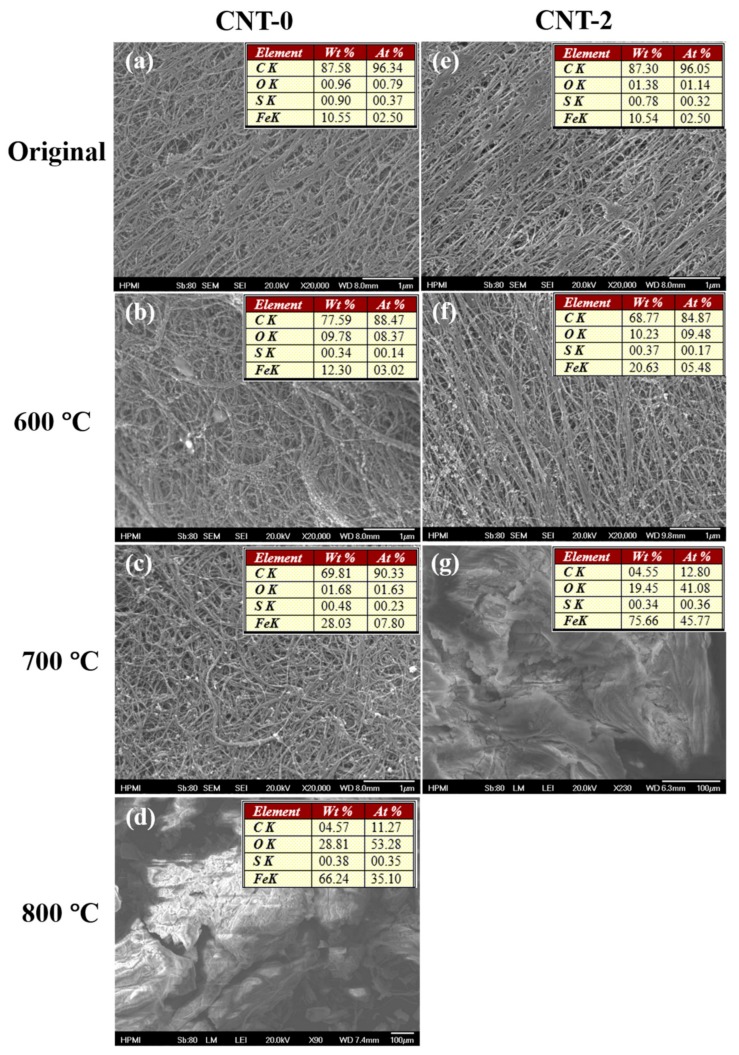
SEM images and corresponding energy-dispersive X-ray spectroscopy (EDS) results showing in tables of oxidized samples taken at different temperatures: (**a**–**d**) CNT-0 samples and (**e**–**g**) CNT-2 samples.

Interestingly, an increasing trend of the catalyst residue (weight percentage at 800 °C) was observed as the water concentration increased (18.57 wt.%, 22.86 wt.%, and 23.89 wt.% for CNT-0, -1, and -2, respectively). To better understand this trend, CNT-0 and -2 samples were taken at 600 °C, 700 °C, and 800 °C during the TGA tests and examined using SEM and EDS- the results are displayed in Figure 4. For the as-synthesized samples, the EDS results indicate a similar catalyst residue starting point at 10.55 wt.% and 10.54 wt.% for CNT-0 and CNT-2 groups, respectively, presumably due to the identical synthesis conditions except for water concentration. At 600 °C (~580 °C of the sample), the relative Fe content of the water-assisted (CNT-2) group increased to 20.63 wt.%, suggesting the initiation of carbon oxidation corresponded with the first derivative weight peak in the TGA curve, while that of the regular (CNT-0) group remained relatively constant (10.55 wt.% to 12.30 wt.%). The discrepancy was further magnified by 700 °C (~680 °C of the sample), when the CNT-2 group accomplished the oxidation. The oxidation of the CNT-0 group was not complete until 800 °C (~780 °C of the sample). The earlier oxidation of the water-assisted CNT-2 samples could be attributed to their smaller diameters, resulting from the removal of an aC layer on the catalyst nanoparticles before the CNT growth. Furthermore, the resultant reduction in size of the Fe nanoparticles could also allow for lower temperature oxidation [32]. 

### 3.2. Morphology Analysis

The SEM images shown in Figure 4 provide an overview of the sample surface morphology, where the nanotubes entangled together presenting a distinct bundle network structure, and the bundles are typically long with a uniform diameter distribution. During the collection process, the rotating spindle induced a stretching force that slightly aligned the network. This alignment has been reported to improve CNT bundle packing [33,34], hence the mechanical and electrical properties, which could benefit multifunctional composite fabrications.

**Figure 5:**
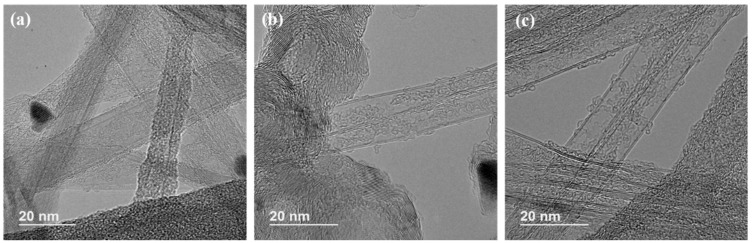
TEM images of the as-produced FCCVD-CNTs: (**a**) CNT-0, (**b**) CNT-1, (**c**) CNT-2, respectively.

The internal structures of all three CNT samples were revealed with HRTEM (Figure 5). For regular-catalyst-based CNTs (CNT-0, shown in Figure 5a), the predominant component observed was MWCNTs with diameters ranging from 19 to 23 nm. The addition of de-ionized water into the catalytic solutions transitioned the composition towards more DWCNTs, where DWCNTs started to emerge (Figure 5b) in the CNT-1 samples, accompanying some MWCNTs with more than ten walls. With an even higher water concentration, the CNT-2 group demonstrated a great majority of DWCNTs, with diameters ranging from 10 to 15 nm. These findings were in good agreement with the TGA derivative peak shifting to lower temperatures (Figure 3), as a result of the decreasing CNT wall counts and diameters. The morphological changes confirmed our earlier hypothesis that water selectively reacted with and removed the amorphous carbon coating on the Fe nanoparticles without a significant etching effect on the nanotubes. This resulted in the reduction of catalyst sizes, therefore the synthesized CNTs with smaller diameters and decreased wall counts.

### 3.3. Raman Spectroscopy

**Figure 6:**
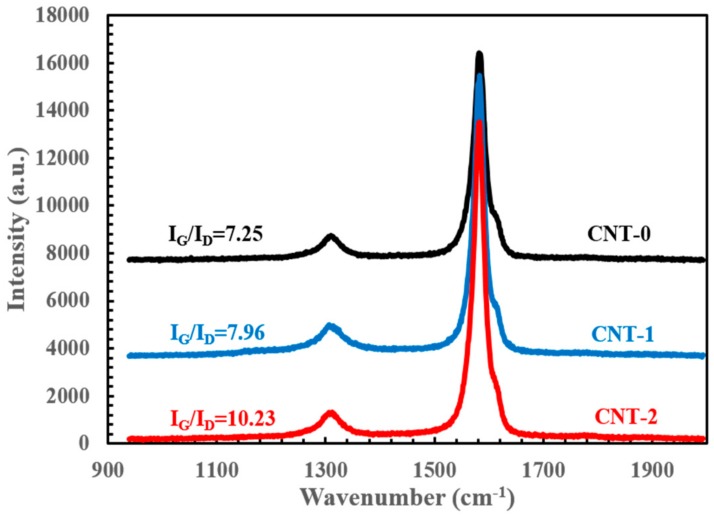
Raman spectroscopy comparing the regular and water-assisted FCCVD synthesis with I_G_/I_D_ values labelled.

Raman spectroscopy analysis was conducted to study the resultant CNT quality by the means of detecting the sp^2^ and sp^3^ hybridized carbon [35]. Figure 6 shows a strong G-band at approximately 1585 cm^−1^, which is characteristic of ordered graphitic structure and corresponds to the tangential vibration of carbon atoms along the CNT axis [36]. Additionally, a disorder-induced D-band at around 1310 cm^−1^ indicates the relative amount of the defective impurities or other symmetry-breaking defects, especially amorphous carbon in the case of as-produced FCCVD-CNTs [37]. Therefore, the intensity ratio of the above-mentioned peaks (i.e., I_G_/I_D_) could be used as a direct measure of CNT quality.

In the CNT-0 group, the I_G_/I_D_ ratio was calculated to be 7.25, which indicated good CNT quality [36]. Furthermore, the G-band feature exhibited an asymmetric characteristic line shape with a weak peak appearing at ~1590 cm^−1^. This feature was likely associated with the large amount of multi-walled carbon nanotubes, due to the diameter variations among different nanotubes in a typical as-collected CNT assembly [38]. Under identical experimental conditions, the addition of a small amount of de-ionized water increased the I_G_/I_D_ ratio to 7.96 and 10.23 for CNT-1 and CNT-2 groups, respectively. This CNT quality improvement could possibly be attributed to the effectiveness of catalyst cleaning by amorphous carbon removal. Note that the G-band asymmetric feature became less conspicuous as the water concentration increased, suggesting a narrower diameter distribution of MWCNTs with fewer disorders. Meanwhile, no radical breathing mode (RBM) peaks were observed from the collected Raman spectra (not shown here). As a result of the linear correlation between the RBM frequency and the inverse of nanotube diameter [36,39], the MWCNTs of wide diameter distribution may have smeared out the RBM signals from the increased amount of DWCNTs. 

### 3.4. Mechanical Properties

Through resin spraying and standard curing processes as previously reported [40], the CNT-0, -1, and -2/bismaleimide (BMI) composites were fabricated, and the CNT contents were calculated to be comparable at 53%, 57%, and 57%, respectively. To evaluate the mechanical properties, tensile tests were performed on the as-collected CNT thin films as well as the corresponding CNT/BMI composites, and they were summarized in Table 1. Substantial differences between the axial and transverse directions among all the groups can be explained by the stretching-induced alignment during collection. Despite the macroscopic defects intrinsic of the as-collected CNT thin films due to the lack of post-treatment steps (i.e., densification), mechanical improvements in both tensile strength (σ) and Young’s modulus (E) were significant. With the addition of 1 mL water, the tensile strength and Young’s modulus of CNT-1 sheets were 284 ± 75 MPa and 7.8 ± 2.3 GPa, respectively, which were 26% and 47% higher than those of the CNT-0 group. The increment was further increased to 67% and 92% as the DI water usage was doubled, which set the tensile strength and modulus at 473 MPa and 15.0 GPa, respectively, for CNT-2 sheets.

**Figure 7:**
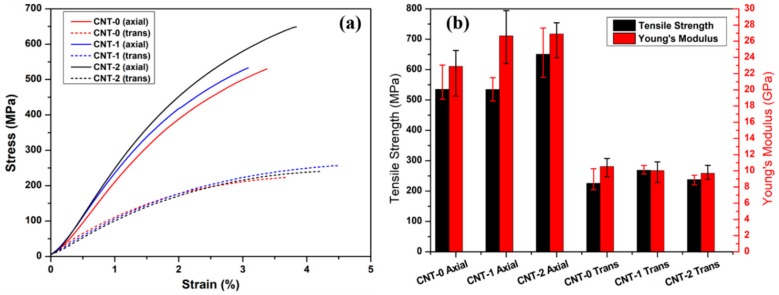
Mechanical properties of the CNT/BMI composites using regular (CNT-0) and water-assisted (CNT-1, -2) FCCVD synthesis: (**a**) typical tensile stress–strain curves and (**b**) ultimate tensile strength and Young’s modulus.

Figure 7 shows the typical tensile results along the axial and transverse directions. Along the alignment (longitudinal) direction, the CNT-0/BMI and CNT-1/BMI composites demonstrated similar tensile strengths of 534.8 ± 80.1 MPa and 534.4 ± 39.0 MPa, respectively. A 21% increment in tensile strength was observed for the CNT-2/BMI composites. The Young’s modulus of the water-assisted FCCVD-CNT (-1, -2)/BMI composites were 26.65 ± 3.40 GPa and 26.90 ± 2.94 GPa, respectively, which was 16% higher than that of the CNT-0/BMI samples. The improved mechanical properties could possibly be accredited to the decreased number of CNT walls and smaller diameters (i.e., DWCNTs) [41]. Additionally, less aC with the water cleaning effects may have enhanced the CNT/BMI interface. The transverse tensile stress–strain curves were found to be close to each other, due to the weak intermolecular bonding.

### 3.5. Electrical Conductivities

**Figure 8:**
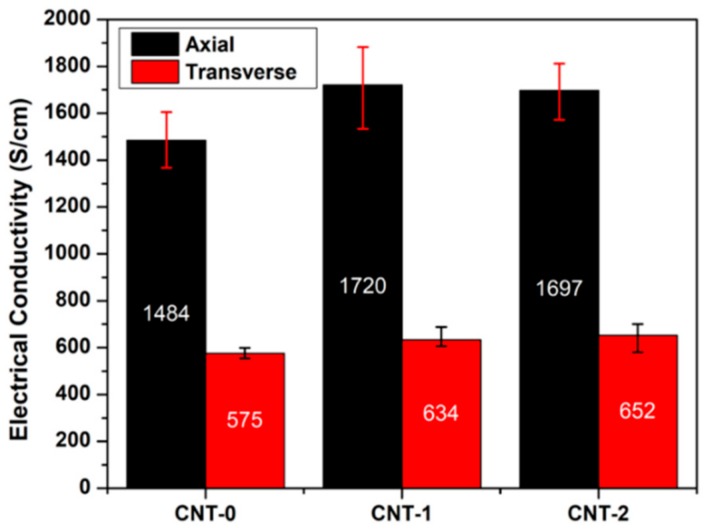
Electrical conductivities of the CNT/BMI composites.

Stemming from the unique electronic structure of two-dimensional graphene, individual SWCNTs and MWCNTs could possess electrical conductivity as high as 10^6^ S/cm [42] and 3 × 10^4^ S/cm [43], respectively. However, macroscopic assemblies (e.g., yarns, sheets) typically demonstrate much lower values because of impurities, CNT discontinuity, and misalignment [44], which limit their potential applications as lightweight conductors [45]. Electrical conductivities of the as-collected CNT films and the manufactured CNT/BMI composites at different water concentrations were measured and presented in Figure 8 and Table 1. Similarly, substantial differences in the electrical conductivity were found when measured in the longitudinal or transverse directions—once again indicating partial CNT alignment. In general, the as-collected CNT thin films demonstrated electrical conductivities of 885 S/cm, 1473 S/cm, and 1820 S/cm, as more water was added into the system. These extraordinary electrical properties confirmed the high quality of the collected CNTs by being highly comparable to those of the mechanically densified FCCVD-CNT films reported [46,47,48], possibly due to a larger DWCNT proportion with fewer defects. During the process of composite manufacturing, the electrical conductivities of CNT-0, and -1 groups were further enhanced, as new electrical pathways might have been built in the through-thickness direction with additional densification. In the composite group, both water-assisted groups—CNT-1/BMI and CNT-2/BMI—showed higher conductivities at 1720 ± 186 S/cm and 1697 ± 125 S/cm, respectively, which was a ~16% increase referenced to the CNT-0/BMI composites. Interestingly, both mechanical and electrical properties of the CNT/BMI composites exhibited a consistent anisotropy ratio around 3, which is presumably an indicator of the stretching-induced CNT alignment.

### 3.6. Mechanical Properties from Nano-Indentation Tests

**Figure 9:**
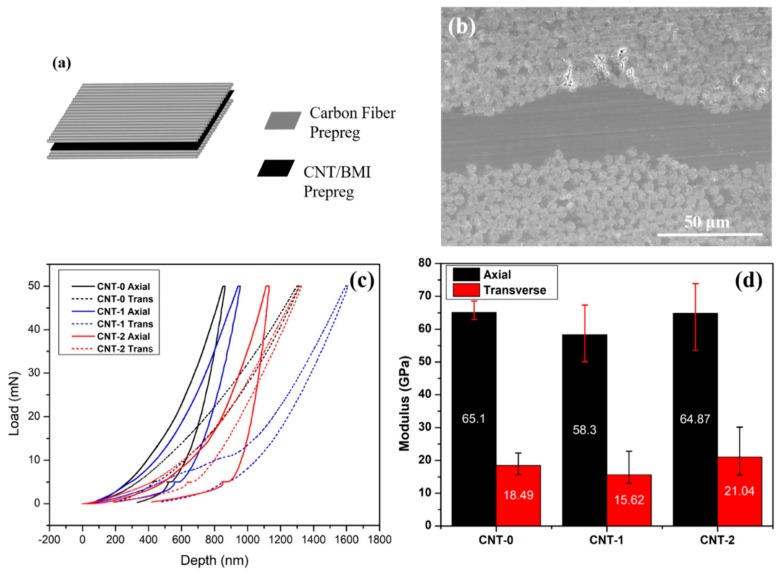
Nano-indentation tests: (**a**) illustration of the CF/CNT/BMI hybrid composites for the test, (**b**) cross-sectional morphology of the hybrid composites showing the CNT sandwiched in between CF prepregs, (**c**) typical load–depth curves, and (**d**) the reduced moduli of the CNT-0, -1, -2/BMI composites along longitudinal and transverse directions.

To further study the mechanical properties of the resultant nanocomposites, nano-indentation tests were conducted to reveal more detailed microstructure–property relationships [20,21] and provide nanoscale insights of elastic modulus and indentation hardness. Figure 9 presents the typical load–depth curves and the calculated reduced moduli of the CNT/BMI composites at different water concentrations. It is evident that the modulus increased significantly (by 100%–150%) compared to the tensile Young’s modulus shown in Figure 7b, due to the differences between the tensile and nano-indentation test methods. The higher modulus from nano-indentation could be originated from the characteristics of CNT composites in that the tensile properties are more sensitive to the load transfer between CNT and resin matrix than compressive properties reflected in the nano-indentation tests [49]. The modulus improvement could also be attributed by the fact that the nanoscale characterizations exclude the influence of discontinuous CNT bundles. Interestingly, the anisotropy ratios of longitudinal and transverse direction moduli from the nano-indentation of CNT/BMI composites were also ~3, consistent with those from the tensile and electrical conductivity measurements.

## 4. Conclusions

Continuous synthesis and collection of CNTs based on the FCCVD process were performed with various catalyst modifications. Instead of mixing water vapor with carrier gas, different amounts of de-ionized water were added as an additional oxidizer to the conventional catalyst feedstock with ferrocene, thiophene, and acetone. Compared to the control sample, the addition of water selectively removed aC and reduced the amount of MWCNTs, yielding more DWCNTs. As the collisions and agglomerations among the iron nanoparticles are almost impossible to control, the as-collected samples from FCCVD process are still mixtures of different types of CNTs (i.e., different wall counts) with wide diameter distribution. TGA results and TEM images showed that CNTs synthesized with the addition of water in the catalyst solution exhibited reduced diameters and improved quality, as confirmed by the higher I_G_/I_D_ ratio from Raman spectroscopy.

Tensile tests on the original CNTs and corresponding CNT/BMI composites showed improved properties. The CNT-2/BMI composites exhibited a maximum longitudinal tensile strength of 650.2 MPa and Young’s modulus of 26.90 GPa, which were 21% higher than that of the CNT-0/BMI composites synthesized from regular catalyst conditions, i.e., without the addition of water. Additionally, the longitudinal electrical conductivities of the CNT-2/BMI composite (~1700 S/cm) was 16% higher than the CNT-0/BMI group. Both results indicated that water cleaning resulted in improved CNT quality and CNT/BMI interface with reduced aC. Interestingly, the nano-indentation tests on the CNT/BMI composite showed similar anisotropy ratios of ~3 as observed in tensile and electrical properties. The improved quality of water-assisted FCCVD-CNTs with enhanced mechanical and electrical properties could further facilitate CNT application studies.

## Figures and Tables

**Table 1:** Mechanical and electrical properties of the CNT-0, -1, -2 thin films and the corresponding CNT/bismaleimide (BMI) composites.

	Tensile Strength (MPa)			Young’s Modulus (GPa)			Electrical Conductivity (S/cm)		
	Axial	Trans	T_//_/T_⊥_	Axial	Trans	E_//_/E_⊥_	Axial	Trans	σ_//_/σ_⊥_
CNT-0	225 ± 43	44 ± 15	5.11	5.3 ± 1.2	1.5 ± 0.3	3.53	885 ± 77	876 ± 22	1.01
CNT-0/BMI	515 ± 15	205 ± 21	2.51	23.4 ± 1.3	9.8 ± 0.9	2.39	1484 ± 121	575 ± 24	2.58
CNT-1	284 ± 75	83 ± 22	3.42	7.8 ± 2.3	2.7 ± 1.2	2.89	1473 ± 61	1455 ± 99	1.01
CNT-1/BMI	534 ± 39	252 ± 26	2.12	27.9 ± 1.8	9.8 ± 1.3	2.85	1720 ± 186	634 ± 54	2.71
CNT-2	473 ± 46	64 ± 13	7.39	15.0 ± 3.9	2.7 ± 0.9	5.56	1820 ± 67	1727 ± 71	1.05
CNT-2/BMI	497 ± 78	235 ± 18	2.11	21.2 ± 2.8	9.5 ± 0.5	2.23	1697 ± 125	652 ± 71	2.60
